# Teleconsultations in oral medicine: dentist perception during the COVID-19 pandemic in Brazil

**DOI:** 10.4317/medoral.26456

**Published:** 2024-04-14

**Authors:** Keyla Marinho de Paiva, Samuel Trezena, Denise Maria Mendes Lúcio da Silveira, Lucas Alves Trindade de França, Daniella Reis Barbosa Martelli, Antônio Luiz Pinho Ribeiro, Paulo Rogério Ferreti Bonan, Hercílio Martelli-Júnior

**Affiliations:** 1 State University of Montes Claros (Unimontes), Biological and Health Sciences Center, Dentistry School, Montes Claros, MG, Brazil; 2 Department of Health of the State of Minas Gerais (SES-MG), Regional Superintendence of Health of Montes Claros, Montes Claros, MG, Brazil; 3 Federal University of Minas Gerais (UFMG), Medicine School, Department of Medical Clinical, Belo Horizonte, MG, Brazil; 4 Federal University of Paraíba (UFPB), Health Sciences Center, Department of Clinical and Social Dentistry, João Pessoa, PB, Brazil

## Abstract

**Background:**

The aim of this study was to describe the perception of dentists from the North macroregion of Minas Gerais, Brazil, users of telediagnosis in Oral Medicine, during the COVID-19 pandemic.

**Material and Methods:**

This is a cross-sectional and descriptive study. Data collection was carried out online, between May and October 2022. The information was transferred to the Statistical Package for the Social Sciences for Windows (SPPS)® version 24.

**Results:**

The sample consisted of 255 dentists, predominantly female. Regarding perception, a significant percentage (47.8%) of respondents agreed that they would like to use telediagnosis frequently, more than half (60.6%) agreed that the technology is easy to use, only a small percentage (8.8%) needed technical support to use it and almost half (48.2%) mentioned the desire to continue using it after the pandemic. When asked if patients felt confident and comforTable when passing on information, more than half disagreed or remained neutral (58.4%), a similar result was found in relation to confidence in the application of the instrument by professionals.

**Conclusions:**

It is concluded that, during the pandemic, telediagnosis in Oral Medicine was an easy and adequate tool. However, professionals must be trained and prepared to be comforTable and ready for use.

** Key words:**COVID-19, oral medicine, telemedicine, teledentistry.

## Introduction

In early 2020, the World Health Organization declared the spread of COVID-19, caused by SARS-CoV-2, a pandemic (1). COVID-19 is highly contagious (2), and its transmission can occur during clinical dental practice due to the production of aerosols with exposure to contaminated droplets (3-5). Due to the advances of COVID-19, the routine of dental services was significantly affected and elective care was suspended, leaving only urgent and emergency care to be provided (6).

Due to the COVID-19 pandemic, health practices needed to undergo significant changes, with new care models encouraged, such as the use of telemedicine or telehealth. These tools use technological communication resources to promote health and improve access, quality, and efficiency at a geographic distance (7). During periods of social isolation, the use of telehealth can be an important coping mechanism, as it enables better continuity of care (5,8,9).

Likewise, to ensure the adequacy of dental practice in the face of COVID-19, in June 2020, the Federal Council of Dentistry (CFO in Portuguese) published resolution 226/2020, which allowed teledentistry, teleguidance, telemonitoring, within the scope of the Unified Health System (SUS in Portuguese) (6). During the pandemic, teledentistry primarily enabled public services to carry out telemonitoring to monitor priority groups and facilitate early diagnosis. However, it also enabled the remote tracking of suspected cases and the ability to identify those that required a face-to-face consultation (5).

In addition, teledentistry was widely used during this period for teleconsulting and telediagnosis, in which general dentists could share information with specialists (5). Amid the COVID-19 pandemic, the oral telediagnosis service has proven to be a reliable method a represents a promising alternative for the clinical support of health professionals, especially in areas with limited access or healthcare services (10). Reports from China (11) and the United States (12) have described good adherence to these tools by health professionals, which have been used to provide guidance and follow up to patients with oral lesions with potential malignancy. In Brazil, reports on services and case reports in which information and communication technologies (1,13-15) were used in the management and care, and the use of smartphones was considered the gold standard in the diagnosis of cases with a perfect coefficient alpha, have been published (7). However, the knowledge of dentists about the application of teledentistry remains low (16).

Although telehealth has the potential to improve the quality of care offered in the public healthcare system and despite advances in teledentistry modalities, it is necessary to evaluate the perception of dentists’ about these services. Thus, this study aimed to describe dentists' perception with an oral medicine telediagnosis service used during the COVID-19 pandemic.

## Material and Methods

- Participants and study setting

This was a quantitative cross-sectional study conducted with dentists who worked in Primary Health Care (PHC) in the North Health macroregion of the state of Minas Gerais, Brazil. The region is one of the 13 health macroregions of the State Secretariat of Minas Gerais (SES in Portuguese), Brazil. It is characterized by limited social indicators, significant social inequality, and a widespread population with low density. These characteristics include a population that depends almost exclusively on PHC services, and that suffered from the suspension of dental care offered by municipalities and reference services (8).

- Telediagnosis service in oral medicine

In view of the pandemic situation and in compliance with the recommendations for standardizing dental care in public health services, on April 20, 2020, a Whatsapp channel was created by the Oral Telediagnosis Program in Stomatology and Oral Pathology developed through the partnership established between the State University of Montes Claros- Minas Gerais- Brazil, through the Department of Dentistry, and the Minas Gerais Telecare Network. This service aimed to serve the northern macro-region of the state of Minas Gerais, with the aim of providing remote advice to dentistry professionals for diagnosis, guidance and clinical treatment of oral lesions. Stomatology specialists, through this teleconsultation tool, remotely guided primary care professionals on the management of identified oral lesions.

- Sampling

Initially, a survey was carried out on the number of dentists registered in the public health service in the National Register of Health Establishments (CNES in Portuguese), considering all levels of care, totaling 739 professionals. The sample size was calculated based on the number of professionals registered at the CNES, considering an estimated prevalence of 50%, with a margin of error of 5% and a confidence interval of 95%. Based on the adopted criteria, the survey needed at least 253 participants. Professionals working in PHC in municipalities that comprise the northern macroregion of Minas Gerais were considered eligible. Professionals who were not working as dentists in the first year of the pandemic, did not use teleconsulting resources in oral medicine, and were on vacation or away from work during the data collection period were excluded.

- Data collect

Data were collected online through a questionnaire designed and administered by the researchers, using the Google Forms platform. It was made available digitally through an instant messaging application (WhatsApp®) between May and October 2022. Of the 329 respondents, 255 met the inclusion criteria and were included in the analysis. During the telediagnosis service during the pandemic, 287 teleconsultations were conducted.

The questionnaire consisted of 16 questions related to the use of and perception with oral medicine telediagnosis services during the COVID-19 pandemic. A brief introduction, consisting of an invitation to participate and a brief summary of the study, confirming that all information collected would remain confidential, was shared with the participants before they started the questionnaire. The following information was obtained: sex, workplace changes during the pandemic, the diagnostic purpose of using the teleservice, the biggest problem in their work routine faced during the pandemic, the teleconsulting method used, their desire to use technology frequently, the ease of use of the telehealth service, the presence of problems and need for technical support, their professional confidence in the use of technology, and patient comfort and desire to continue using such technology after the pandemic.

- Data analysis

The collected data were entered into the Statistical Package for the Social Sciences for Windows (SPPS)® version 24.0 to build a database and perform statistical analyses. This study was approved by the Institutional Ethics Committee (#5.267.570), is in compliance with the Helsinki Declaration and that each participant had access to a detailed informed consent form.

## Results

The final sample was made up of 255 dentists who used the telediagnosis service during the pandemic. The vast majority of professionals were female (*n*=191; 74.9%) and reported workplace changes during the pandemic (*n*=224; 87.8%). The main reasons for using teleservices for diagnostic purposes were: following up patients (*n*=207; 57.8%), discussing cases with other dentists (*n*=64; 17.9%), and evaluating whether a patient needed to be seen face-to-face (*n*=87; 24.3%). Regarding the teleconsultation method used, 61.3% of professionals (*n*=209) used telephone calls, and 38.7% (*n*=132) used WhatsApp (Table 1).

Regarding perception, a significant percentage of respondents agreed that they would like to use telediagnosis frequently (*n*=108; 47.8%), and more than half of the dentists agreed that the technology was easy to use (*n*=137; 60.6%). Only a small percentage (*n*=20; 8.8%) needed technical support, and almost half mentioned the desire to continue using it after the pandemic (*n*=109; 48.2%). However, when asked if the patients felt confident and comforTable when passing on the information, more than half disagreed or remained neutral (*n*=132; 58.4%). A similar result was found regarding confidence in the application of telehealth by professional users (*n*=133; 58.8%). More information on the factors related to dentists' satisfaction is presented in Table 2.

The oral changes, for which dentists most sought the teleconsultation service, were red, single lesions, with regular contours, smooth surface, sessile implantation base, measuring approximately 0.5 to 1 centimeter, with the presence of some discomfort and absence of bleeding. The average time for professionals to use the service, enough to pass on all the information, was 15 minutes for each teleconsultation, with the specialist's response being almost immediate.

Discusson

The teleconsulting service was a tool adopted by dentists in the North macroregion of Minas Gerais, Brazil, during the pandemic, with more than half using it to follow up patients. Moreover, the current study found that a significant percentage of dentists would like to continue using the teleconsulting service. During the social isolation period of the COVID- 19 pandemic, the adoption of information technologies helped to increase the responsiveness of the health system, allowing access to professional guidance by patients (8). The current study reviewed the use of an important telehealth tool that a higher education institution developed as a reference for diagnosing and treating oral lesions and maintaining patient follow up.

The present study found that a significant percentage of dentists reported changes to their work routine during the pandemic, including the use of telehealth. This demonstrates compliance with the recommendations implemented by the Ministry of Health, which regulated dental care in the SUS in accordance with CFO guidelines in accordance with local epidemiological characteristics in the face of the COVID-19 pandemic, such as the use of telehealth modalities (6).

Due to the COVID-19 pandemic in Brazil, dental care was restricted to urgent and emergency care to minimize the risk of exposure of dentists to the virus and prevent cross- infection. As a result, the pandemic drastically reduced the number of cancer diagnoses, probably due to the restrictive measures established (17). This also significantly impacted the number and type of oncological surgeries being conducted (18). In this context, the present study shows that the use of the teleodontology tool was valuable for early diagnosis, as well as in monitoring and treatment cancer.

Teledentistry can be widely applied in the field of oral medicine, especially in events such as the COVID-19 pandemic, as it can help to prevent increased morbidity of various oral diseases (1). Long-distance communication between professionals in PHC and oral medicine specialists through the use of teleconsultations can help speed up the diagnosis of patients and provide immediate referrals when necessary (9). A survey conducted to describe the dental care offered by the SUS during the COVID-19 pandemic showed that local oral health services had to adapt their practices to reduce the spread of COVID-19, with teleservice tools being used along with the installation of sanitary barriers (8).

A study conducted in Canada analyzed how Canadian organizations supported teledentistry and found an increase in the use of this modality in all regions (19). Teledentistry, through the use of photographic teleconsultations, was implemented during the pandemic by the Department of Surgery and Oral Pathology of Magna Grecia University, Italy, both for urgent consultations and to promote the continuity of treatments, such as post-surgical evaluations with promising results (3).

Regarding perception of the teleconsulting tool, the present study found that 47.8% of the respondents would like to use the system frequently, and more than half found that the system was easy to use. Moreover, a significant percentage reported that they would continue using the teleconsulting tool even after the pandemic. However, the current study also found that a significant percentage of professionals disagreed or remained neutral when asked if they felt confident about using teleservices.

In the state of Paraná, Brazil, a study that analyzed an asynchronous telediagnosis service of oral lesions through Telessaúde Brasil Redes reported that the program was generally favorably evaluated by professionals and that the service fully met the needs of the professionals for 92% of requests, thus avoiding, for the most part, unnecessary referrals to specialist services (9). In a study that evaluated the impact of the use of teledentistry and its trends in dental practice with professionals from several countries, more than half of them agreed that teledentistry was a useful tool to improve clinical practice, and most would recommend its use in oral medicine (20). There is evidence that teledentistry is considered a low- cost and accessible modality of care, as it brings together professionals from geographically distant regions, is of great importance for public health services, reduces inequalities, and promotes equity (20). Furthermore, it can help to diagnose some conditions earlier, which is particularly important for potentially malignant lesions (10).

A study conducted during the pandemic with dentists from different regions of Brazil aimed to assess their level of knowledge, perception, and experience with teledentistry services. In contrast to many studies, they concluded that the majority of participating dentists had never used teledentistry resources, felt unprepared and insecure, and that patients would have some difficulty accepting it as part of dental care (16). Other studies have identified barriers to the adoption of teledentistry by professionals, such as resistance to new technologies, complexity, and a lack of continuous technical support (19,21). However, the current study found that 96.9% of the respondents disagreed that the system was too complicated to use, and only 8.8% required technical support.

In the United States, a study that explored the perception of dentists regarding the use of teledentistry highlighted that despite cautious optimism and an increase in interest in teledentistry by some professionals during the pandemic, some professionals did not adopt the technology for ethical reasons and/or doubted the quality of care provided (22). Similar results were found in studies that identified difficulties in obtaining an accurate diagnosis through teledentistry as a reason for the lack of its acceptance by dentists, and highlighted the lack of resources for the correct storage of patient information and the need for technical support (5,23).

Considering the perspective of patients in relation to teledentistry, the present study found that approximately 41.6% of the responding dentists thought that their patients were comforTable and confident when passing on information via the telehealth service. One study pointed out that 91% of patients were able to understand the use of the tool and maintain good communication with their dentists, as if they were face-to-face, with 97% satisfaction (24). Another study conducted during the COVID-19 pandemic reported that 100% of patients were satisfied with an oral medicine teledentistry service (1).

In the present study, telephone calls (61.3%) and WhatsApp (38.7%) were used for telehealth consultations. Teledentistry can be performed through several platforms, such as instant messaging applications (WhatsApp, Telegram, Messenger), videoconferencing applications (Google Meet, Skype, Facetime), and social media (24). WhatsApp and Facebook are the applications most used by doctors and medical students from hospital institutions in Minas Gerais, and a study confirmed that telehealth tools are becoming increasingly popular in health (25). In a previous study, videoconferencing was the preferred method, followed by telephone (22). The use of WhatsApp for the management of oral medicine patients was reported to have significant success in Rio Grande do Sul, Brazil (26). Another study conducted with several dentists from different regions of Brazil found that most preferred using video calls and text messages for digital consultations (16). In a study of Australian dentists, telephone and email were the preferred means of communication (23).

This study has some limitations. First, data collection was conducted remotely, which could result in some missing data. In addition, some professionals do not have technological skills and, consequently, could not easily access the questionnaire link. Another limitation is the lack of knowledge among dentists regarding teledentistry and its modalities of assistance in oral health. Convenience sampling due to the pandemic is another identified limitation. However, this study was conducted in a region with many disparities in access and a population that depends almost exclusively on public services. This study has identified which teledentistry modalities were used and that a significant number of surgeons were actively responding to dentists.

## Conclusions

During the COVID-19 pandemic, technological resources were widely used to reduce geographical barriers, improve access and ensure continuity of care. It was evident in the current study that telehealth is easy to use and adequate in many cases. The implementation of this service as an auxiliary tool for remote diagnosis of oral lesions in dentistry, even after the end of the pandemic, could help to facilitate better access to health services. Professionals must be trained in telehealth to ensure they can be more confident using the available technological tools and manage patient information safely and correctly.

## Figures and Tables

**Table 1:** Use of teletechnology during the pandemic by dentists in the North macroregion - Minas Gerais - Brazil (n=255).

Variables		Descriptive n (%)
Sex	Male	64 (25.1)
	Female	191 (74.9)
Changes in the workplace	Yes	224 (87.8)
	No	31 (12.2)
Biggest problem during quarantine	Patient absences	77 (20.5)
	Lack of material	38 (10.1)
	Opening restrictions	76 (20.3)
	Insecurity	179 (47.7)
	Locomotion problems	5 (1.3)
Virtual query method used	Phone call	209 (61.3)
	Messaging apps	132 (38.7)
Diagnostic purpose	Patient follow-up	207 (57.8)
	Discussing cases with other dentists	64 (17.9)
	Evaluation of the possibility of personal scheduling	87 (24.3)

**Table 2:** Perception of dentists in the North macro-region regarding the use of technology during the pandemic - Minas Gerais - Brazil (n=226).

Variables		Descriptive n (%)
Desire to use technology frequently*	I agree	108 (47.8)
	Neutral, I disagree	118 (52.2)
Ease of use*	I agree	137 (60.6)
	Neutral, I disagree	89 (39.4)
Presence of problems and need for technical support*	I agree	20 (8.8)
	Neutral, I disagree	206 (91.2)
Found the system too complicated to use*	I agree	7 (3.1)
	Neutral, I disagree	219 (96.9)
Patient felt comfortable and confident in passing on all the information*	I agree	94 (41.6)
	Neutral, I disagree	132 (58.4)
Did you feel confident with the application of technology*	I agree	93 (41.2)
	Neutral, I disagree	133 (58.8)
I would like to continue using technology after the pandemic*	I agree	109 (48.2)
	Neutral, I disagree	117 (51.8)

* Variation in the number of 255, due to lack of information (missing data).
